# How Genetics Might Explain the Unusual Link Between Malaria and COVID-19

**DOI:** 10.3389/fmed.2021.650231

**Published:** 2021-04-26

**Authors:** Marta Rusmini, Paolo Uva, Antonio Amoroso, Manlio Tolomeo, Andrea Cavalli

**Affiliations:** ^1^Computational and Chemical Biology, Italian Institute of Technology, Genova, Italy; ^2^Istituto di Ricovero e Cura a Carattere Scientifico (IRCCS) G. Gaslini, Genova, Italy; ^3^Department of Medical Sciences, University of Turin, Turin, Italy; ^4^Department of Health Promotion Sciences, Azienda Ospedaliera Universitaria Policlinico Paolo Giaccone, Palermo, Italy; ^5^Department of Pharmacy and Biotechnology, University of Bologna, Bologna, Italy

**Keywords:** malaria, COVID-19, genomics, whole-genome sequencing analysis, epidemiology

## Abstract

Severe acute respiratory syndrome coronavirus 2 (SARS-CoV-2)-associated coronavirus disease 2019 (COVID-19) pandemic has been the subject of a large number of studies in recent times. Here, starting from the evidence that in Italy, the areas with the lowest number of COVID-19 cases were those with the highest incidence of malaria in the early 1900's, we explore possible inverse relationships between malaria and COVID-19. Indeed, some genetic variants, which have been demonstrated to give an advantage against malaria, can also play a role in the incidence and severity of SARS-CoV-2 infections (e.g., the ACE2 receptor). To verify this scientific hypothesis, we here use public data from whole-genome sequencing (WGS) experiments to extrapolate the genetic information of 46 world populations with matched COVID-19 data. In particular, we focus on 47 genes, including ACE2 and genes which have previously been reported to play a role in malaria. Only common variants (>5%) in at least 30% of the selected populations were considered, and, for this subset, we correlate the intra-population allele frequency with the COVID-19 data (cases/million inhabitants), eventually pinpointing meaningful variants in 6 genes. This study allows us to distinguish between positive and negative correlations, i.e., variants whose frequency significantly increases with increasing or decreasing COVID-19 cases. Finally, we discuss the possible molecular mechanisms associated with these variants and advance potential therapeutic options, which may help fight and/or prevent COVID-19.

## Introduction

Severe acute respiratory syndrome coronavirus 2 (SARS-CoV-2)-associated coronavirus disease 2019 (COVID-19) has gripped the world in a pandemic, challenging its healthcare infrastructure, economy, and culture. In the last months, the epidemic moved from Europe to other parts of the World, growing very rapidly in Latin America, USA, and India. In Africa, the impact of COVID-19 is worrying the whole world due to the precariousness of the healthcare system with limited financial resources and scarcity of solid infrastructures and trained health workers (1). Additionally, COVID-19 immune-response and symptoms, including fever, fatigue, headache, gastrointestinal issues, etc., are very similar to those of malaria and other infections, which are endemic in many sub-Saharan areas. This may lead to a delayed diagnosis and make the access to health facilities more difficult. However, despite the fear and the alert about sub-Saharan countries, SARS-CoV-2 has not hit malaria-endemic regions so strongly as everyone would have expected (2). These observations prompted us to analyze from the epidemiological and genetic standpoints possible links between malaria and COVID-19.

Previous studies have shown an inverse relationship between the number of COVID-19 cases and cases of malaria (3–6). Three of the African countries most affected by COVID-19 on June 4, 2020 are South Africa (37,525 cases and 792 deaths), Algeria (9,733 cases and 673 deaths) and Egypt (28,615 cases and 1,088 deaths), which belong to the countries historically less affected by malaria (7). This has been reported to be due to prophylaxis with chloroquine and hydroxychloroquine, which however have shown limited efficacy in COVID-19 (8). Furthermore, a recent publication confirmed a lower risk of COVID-19 in malaria-endemic areas and, albeit with an underlying mechanism that has yet to be investigated, they identified shared immunodominant epitopes between SARS-CoV-2 and P. falciparum antigens (9). Against this scenario, here we aim to investigate this inverse relationship, from a genetic point of view.

Humans have been infected by and co-evolved with malaria for over 50,000 years (since the estimated date for the out-of-Africa migration) (10). Different human polymorphisms have been associated with this natural selection process (11). For example, hypertension confers an evolutionary advantage and protection for populations chronically exposed to malaria infections. Epidemiological data indicate that people with African background have a higher prevalence of hypertension compared to whites coming from malaria-free areas (12, 13). Hypertension could actually protect people from developing cerebral malaria, and angiotensin II is the key molecular effector in the hypertension-malaria relationship (14). Angiotensin II levels tend to be higher in populations living in malaria-endemic areas, and preclinical studies have shown that angiotensin II could inhibit the plasmodium growth and erythrocyte invasion (12). The angiotensin-converting enzyme 2 (ACE2) degrades angiotensin II to generate angiotensin 1-7, which acts as a vasodilator. Therefore, low levels of ACE2 cause an increase in angiotensin II and hypertension, and a natural selection process may have produced the association between ACE2 polymorphisms and protection from severe malaria (15).

The ACE2 gene is located on the X chromosome and several single-nucleotide polymorphisms (SNPs) within the ACE2 gene have been reported. For instance, the ACE2 rs2106809 T allele in intron 1 has been associated with reduced expression of the ACE2 enzyme (16). A recent finding has shown that the “D” allele of ACE I/D polymorphism, responsible for increased angiotensin II production, has a significant association with mild malaria, and the ACE2 rs2106809 T allele has gender specific effect with reduced expression of ACE2 in presence of “T” allele in women. This leads to increased level of angiotensin II and hence protection against severe, cerebral malaria (15). Remarkably, it has clearly been demonstrated that ACE2 is the major host cell receptor responsible for introducing SARS-CoV-2 into the human body (17).

The observations on ACE2 polymorphism suggest that a genetic predisposition could be worth to be investigated with the final objective to discover other common variations in the genome that could explain the low incidence of COVID-19 in areas where malaria is endemic. Indeed, while SARS-CoV-2 has infected humanity for only less than a year, for tens of thousands of years humans have been confronted with the pathogens responsible for malaria, and therefore natural selection has promoted the genetic variants of greater resistance to the disease.

Based on these preliminary observations, here we first evaluate whether the spread of SARS-CoV-2 infection in the Italian regions is actually different in relation to the historical presence of malaria. Then, we investigate a list of genes linked to positive/negative responses to malaria, using whole genome sequencing (WGS) data from different populations scattered around the world. Only variants with allele frequency (AF) > 5% in at least 30% of the selected populations were considered. For this subset, we subsequently correlate the intra-population allele frequency with the COVID-19 data (cases/million inhabitants) in the first wave (from the beginning of the pandemic to 13th July 2020). In this way, we could identify positive and negative correlations, i.e., variants whose frequency significantly increases with increasing or decreasing COVID-19 cases, hence discovering putative genetic links between malaria and COVID-19. These genetic variants led subsequently us to investigate novel mechanisms, which may help more in-depth understanding the physiopathology and the complexity of COVID-19. Studying these molecular mechanisms, we eventually propose potential novel therapeutic options to combat and/or prevent the spread of SARS-CoV-2 infections.

## Methods

### Datasets

The incidence of malaria at the beginning of 20th century in Italy was reported as the number of deaths for malaria per million inhabitants in 1910, based on the Italian National Report on Causes of deaths (18) and the Italian Historical Statistical Repository (19). In particular, we considered deaths for malaria the total number of deaths for “malarial fever” and “marsh cachexia.” COVID-19 cases for the Italian provinces were obtained from the COVID-19 Italian Data Repository, maintained by the Italian Civil Protection Department (20).

Whole-genome genotyping data of 969 worldwide healthy people from 54 diverse human populations were obtained from the Human Genome Diversity (HGDP) project as VCF files (21). COVID-19 data reported on July 13th, 2020 from the COVID-19 Data Repository at the John Hopkins University (12) were analyzed. Populations with genotyping data were mapped to COVID-19 data according to geographic coordinates available at the HGDP website (22) and at the COVID-19 Data Repository and manually refined as follows: populations with similar coordinates in both repositories were matched. In cases where two populations were geographically very close, the COVID-19 or genetic data were aggregated in order to guarantee a geographical correspondence as reliable as possible. The final set, used for downstream analysis consists of 46 populations for which both data were available for a total of 786 individuals as reported in Table 1.

**Table 1:** Overview of the 46 HGDP populations selected for the study.

**Population**	**Number of individuals**	**Population**	**Number of individuals**
Adygei	16	Mbuti	13
Balochi	24	Miao	10
BantuSouthAfrica	8	Mongolian	9
Basque	22	Mozabite	27
Bedouin	46	Naxi	8
BergamoItalian	11	NorthernHan	10
Biaka	22	Orcadian	15
Bougainville	11	Oroqen	9
Brahui	25	Palestinian	46
Cambodian	7	PapuanHighlands	9
Colombian	7	PapuanSepik	8
Dai	9	Pathan	24
Daur	9	Pima	13
Druze	42	Russian	25
French	28	San	6
Han	33	Sardinian	28
Hazara	19	She	10
Hezhen	9	Surui	8
Japanese	27	Tu	10
Karitiana	12	Tujia	9
Lahu	8	Tuscan	8
Mandenka	22	Yi	10
Maya	21	Yoruba	22

*The number of whole genome sequences available for each population is shown. These populations geographically reside in areas for which the number of COVID-19 cases is available*.

### Time Frame for Selection of COVID-19 Cases

The analysis of Italian provinces and worldwide populations started with COVID-19 data collected in the so-called “first wave,” i.e., from the beginning of the pandemic until mid-July 2020. In order to confirm the results obtained from the “first wave,” COVID-19 cases between 1st November 2020 and 15th February 2021, known as “second wave,” were also considered.

### Genes and Variants Selection

Forty-seven genes were considered in the present study. In particular, in addition to the ACE2 gene we selected (i) all the genes reported in Online Mendelian Inheritance in Man (OMIM) database (23) as related to the susceptibility/resistance to malaria; (ii) genes in known malaria resistance loci (24); (iii) genes with a role in the inflammatory pathway activated by malaria (25) (Table 2). Starting from 67.3 million single-nucleotide polymorphisms from the Whole Genome Sequencing data (21), the selected 47 genes from 46 populations were considered, thus reducing the number of variants to 42,978. Variants were annotated with VEP and custom scripts. The annotation includes the allele frequency of the variants in each of the 46 populations, the allele frequencies in healthy individuals (Gnomad v2.1), the effect of the variants on proteins and the expression Quantitative Trait Loci (eQTLs) data reported on the Genotype-Tissue Expression portal (GTEx Analysis Release V8), showing the effect of each variant in the context of gene expression across different tissues, and the estimates of the effect size (slope coefficient). To pinpoint variants that regulate the transcription of genes, also according to the clinical aspects of COVID-19, only variants with extreme slope coefficients (i.e., outside the interquartile range) in at least four tissues, including lung, were considered at the end.

**Table 2:** List of the genes included in the study.

**Gene**	**Chr**	**Start**	**End**	**OMIM entry**	**Phenotype (OMIM)**	**Reference**
ABO	chr9	133255602	133275214	110300	Blood group, ABO system	(24)
ACKR1	chr1	159203307	159206498	613665	Blood group, Duffy system	(24)
ADORA2B	chr17	15944917	15975746	612446		(24)
ATP2B4	chr1	203626561	203744081	613665	Protection against malaria vivax	(24)
C6	chr5	41142234	41261438	217050		(24)
CD36	chr7	80369575	80674409	173510	Susceptibility to/reduced risk of cerebral malaria	(24)
CD40LG	chrX	136648193	136660390	308230		(24, 25)
CISH	chr3	50606522	50611831	602441	Susceptibility to malaria	
CR1	chr1	207496268	207639409	120620	Resistance to severe malaria	(24, 25)
FCGR2A	chr1	161505430	161518558	146790	Susceptibility to severe malaria	
FCGR2B	chr1	161663161	161678654	604590	Resistance to malaria	
FUT9	chr6	96015974	96215612	606865	Susceptibility to placental malarial infection	
G6PD	chrX	154532000	154547572	305900	Resistance to malaria due to G6PD deficiency	(24)
GNAS	chr20	58841622	58909188			(24)
GYPA	chr4	144109304	144140751	617922	Resistance to malaria	
GYPB	chr4	143996106	144019339	617923	Resistance to malaria	
GYPC	chr2	126656158	126696667	110750	Resistance to malaria	
HBB	chr11	5225464	5227197	141900	Resistance to malaria	(24)
HMOX1	chr22	35381096	35394207			(25)
ICAM1	chr19	10271120	10286615	147840	Susceptibility to cerebral malaria	(24)
IFNa17	chr9	21227243	21228222			(25)
IFNAR1	chr21	33324970	33359864			(25)
IFNb1	chr9	21077104	21077942			(25)
IFNG	chr12	68154768	68159740			(25)
IL10	chr1	206767602	206772494			(24)
IL13	chr5	132658173	132661110			(24)
IL1A	chr2	112773915	112784590			(24)
IL1B	chr2	112829751	112836779			(24)
IL22	chr12	68248242	68253604			(24)
IL4	chr5	132673989	132682678			(24, 25)
IRF1	chr5	132481609	132490773			(24, 25)
LTA	chr6	31572054	31574324			(24)
NCR3	chr6	31588895	31593006	611550	Susceptibility to mild malaria	
NOS2A	chr17	27756766	27800529	163730	Resistance to malaria	(25)
PECAM1	chr17	64319415	64390860			(25)
RNASE3	chr14	20891399	20892348			(25)
SLC4A1	chr17	44248390	44268135	109270	Resistance to malaria	
SPTB	chr14	64746283	64879907			(24)
TGFB2	chr1	218345284	218444619			(25)
TIRAP	chr11	126283087	126294933	606252	Protection against malaria	
TLR1	chr4	38796255	38804791			(24, 25)
TLR4	chr9	117704403	117724735			(24, 25)
TLR5	chr1	223109404	223143248			(25)
TLR6	chr4	38823715	38856817			(24)
TLR9	chr3	52221080	52226163			(24, 25)
TNF	chr6	31575565	31578336	191160	Susceptibility to cerebral malaria	(24, 25)
ACE2	chrX	15561033	15602069			

*Genes are reported with gene symbol, chromosome, start and end genomic position (hg38 coordinates). The MIM number is reported for all the genes associated with malaria in OMIM, and the corresponding phenotype if present. Two other publications were investigated to complete the list, as reported in the last column (“Reference”)*.

### Statistical Analysis

The Spearman's correlation has been used to compute the correlation between COVID-19 cases/10^6^ inhabitants and (i) malaria prevalence in Italy (i.e., number of deaths for malaria/10^6^ inhabitants), (ii) AF of the variants in each population (i.e., fraction of all the alleles in the population that carry the variant). This test assesses the monotonic relationship (whether linear or not) between two variables and is less sensitive to the presence of outliers.

To correct for multiple testing, FDR adjusted *p*-values were obtained using the Benjamini & Hochberg method implemented in *p.adjust* R function. All the analyses were performed in R.

### Linkage Disequilibrium Analysis

Haploview (26) was used to identify variants in Linkage Disequilibium (LD). First, variants in VCF format were converted by vcftools (27) in the input format required by Haploview (26). Only common variants, significantly correlated with COVID19 cases, selected as described above and considered divided for chromosomes, were retained for Haploview analysis, using default parameters. Furthermore, an application of Haploview, named Tagger (28), has been used on all the single nucleotide variants (SNVs) to identify tag SNVs, i.e. SNVs which are representative of a group of SNVs in LD.

## Results

### The Italian Case: The Inverse Correlation Between Malaria and COVID-19

**Figure 1:**
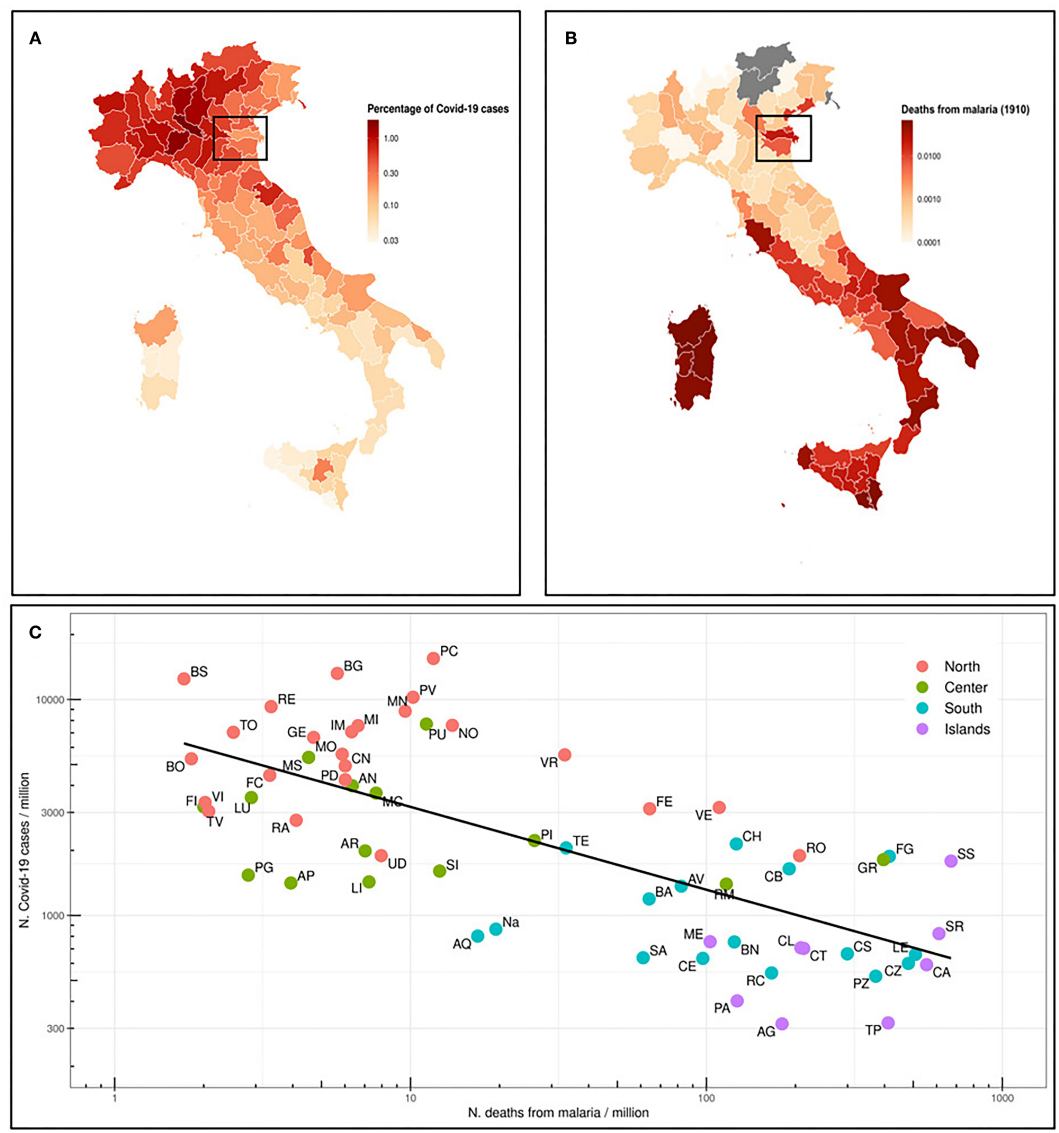
**(A)** Map of the Italian provinces and representation as colorimetric scale of the percentage of deaths for malaria in 1910 (data not available for those provinces that were not included in the Italian Kingdom). The box highlights Ferrara and Rovigo provinces. **(B)** Map of the Italian provinces and the relative percentage of COVID-19 cases as n. COVID-19 cases/10^6^ inhabitants in the province (situation on July 13th, 2020). **(C)** Prevalence of COVID-19 cases (n. cases/10^6^ inhabitants) in Italian provinces (July 13th, 2020) vs. number of deaths from malaria in 1910. The regression line of log10 transformed values is shown. Spearman ρ = −0.69 and *p*-value 5.4e-10.

We first analyzed the situation in Italy, where malaria was endemic until the late 1950's, and therefore prophylaxis strategies have been abandoned several years ago. The Italian regions most affected by malaria were Sardinia, Sicily and other regions in Southern Italy, South Lazio, and the river Po delta (29). In Figure 1A, we represent the percentage of deaths from malaria in 1910 (18) in the current Italian provinces. We also map the percentage of COVID-19 cases as of July 13th, 2020, for the same provinces (Figure 1B) and we correlate both data in Figure 1C. A quite strong inverse correlation (Spearman ρ = −0.69, *p* = 5.4E-10) has been found. Indeed, COVID-19 distribution is clear, with Northern Italy far more affected than Southern Italy. Sicily and Sardinia, along with other regions in Southern Italy, have the lowest number of registered cases, whereas most of Northern Italy is greatly affected by SARS-CoV-2. Interestingly enough, the percentage of COVID-19 cases in Ferrara and Rovigo is much lower than in the rest of the North, and these areas were historically marshy, with a high incidence of malaria (box in Figures 1A,B).

### Genes and Variants Selected

42,978 variants at the 47 genes selected (see Table 2) were used for the analysis. Since the purpose of the study was to identify common susceptibility genetic elements that could help to understand the inverse correlation thus identified, rare variants were discarded. In particular, only 1,411 Single Nucleotide Variants (SNVs) with an allele frequency > 5% in at least 30% of the populations in this study, were considered. Among these, 81 variants strongly correlated (FDR <0.01) with COVID-19 prevalence (n. cases/10^6^ inhabitants) were further investigated and classified based on their positive or negative correlation with COVID-19 cases.

Given the similar allele frequency of many variants throughout the populations, we performed a linkage disequilibrium (LD) analysis to identify blocks of variants in LD and we selected the tag SNVs in LD that could capture the other alleles, leading to a final number of 25 and 16 representative SNVs for positive and negative correlations, respectively (Tables 3A,B). All the genes reported are described in Table 4.

**Table 3:** List of variants with (A) positive and (B) negative correlation (FDR < 0.01).

**rs ID**	**Location**	**Allele**	**Consequence**	**Gene symbol**	**FDR (Spearman)**	**Captured alleles (Tagger)**
**A**						
rs3027012	1:159204333-159204333	T	5' UTR	ACKR1	0.000119	rs3027008, rs6676002
rs72717040	1:161517662-161517662	C	Intronic	FCGR2A	0.004313	rs17400517
rs7683365	4:143999443-143999443	A	Missense	GYPB	0.000161	rs7662277, rs7666297, rs7666296, rs12499907, rs12499906, rs10002395, rs10025455, rs7661933
rs17622656	5:132485305-132485305	A	Intronic	IRF1	0.002085	
rs2770146	9:117711060-117711060	C	Intronic	TLR4	0.000303	rs5030728
rs2285002	14:64816958-64816958	A	Intronic	SPTB	0.005651	
rs12587471	14:64825597-64825597	G	Intronic	SPTB	0.004638	rs11158561, rs4899147
rs7149121	14:64864227-64864227	C	Intronic	SPTB	0.000360	
rs28370916	14:64879376-64879376	G	Intronic	SPTB	0.008414	
rs8081235	17:15964787-15964787	T	Intronic	ADORA2B	0.001541	
rs8081547	17:15965007-15965007	T	Intronic	ADORA2B	0.000487	
**B**						
rs6840234	4:143996657-143996657	C	intronic	GYPB	0.000468	rs6857129, rs4835127, rs1473055, rs6537238, rs4835126, rs6816184
rs8176725	9:133257230-133257230	A	intronic	ABO	0.009139	
rs74056021	14:64802733-64802733	C	intronic	SPTB	0.001538	rs74056022, rs45617438
rs1535450	14:64840590-64840590	T	intronic	SPTB	0.003294	rs1475101
rs28998799	17:27799104-27799105	-	intronic	NOS2	0.000173	
rs4646120	X:15599613-15599613	A	intronic	ACE2	0.006377	
rs1978124	X:15599940-15599940	C	intronic	ACE2	0.001095	

*The first column “rsID” indicates the tag SNVs. “Location” column indicates chromosome and position in the Human Reference Genome Hg38; “Allele” column is the variant allele, “Consequence” column represents the impact of the variant (rsID) on the protein, and the “Gene symbol” is the gene where the variant is located. In “FDR (Spearman)” the FDR value for each SNVs has been reported (data from the first wave). The last column “Captured alleles (Tagger)” displays the rs number of all the SNVs in linkage disequilibrium with the tag SNV, according to Tagger analysis*.

**Table 4:** List of genes with variants significantly (FDR < 0.01) correlated (positively or negatively) to COVID-19 cases.

**Gene symbol**	**Gene name**	**Protein function (Gene Cards)**
ABO	ABO, alpha 1-3-N-acetylgalactosaminyltransferase and alpha 1-3-galactosyltransferase	This gene encodes proteins related to the first discovered blood group system, ABO.
ACE2	Angiotensin I converting enzyme 2	The protein encoded by this gene belongs to the angiotensin-converting enzyme family of dipeptidyl carboxydipeptidases
ACKR1	Atypical chemokine receptor 1 (Duffy blood group)	The encoded protein is the receptor for the human malarial parasites *Plasmodium vivax* and *Plasmodium knowlesi*. Polymorphisms in this gene are the basis of the Duffy blood group system
ADORA2B	Adenosine A2b receptor	This gene encodes an adenosine receptor that is a member of the G protein-coupled receptor superfamily. This integral membrane protein stimulates adenylate cyclase activity in the presence of adenosine.
FCGR2A	Fc fragment of IgG receptor IIa	This gene encodes one member of a family of immunoglobulin Fc receptor genes found on the surface of many immune response cells. The protein encoded by this gene is a cell surface receptor found on phagocytic cells such as macrophages and neutrophils, and is involved in the process of phagocytosis and clearing of immune complexes
GYPB	Glycophorin B (MNS blood group)	Glycophorins A (GYPA) and B (GYPB) are major sialoglycoproteins of the human erythrocyte membrane which bear the antigenic determinants for the MN and Ss blood groups
IRF1	Interferon regulatory factor 1	The encoded protein activates the transcription of genes involved in the body's response to viruses and bacteria, playing a role in cell proliferation, apoptosis, the immune response, and DNA damage response.
NOS2	Nitric oxide synthase 2	This gene encodes a nitric oxide synthase which is expressed in liver and is inducible by a combination of lipopolysaccharide and certain cytokines.
SPTB	Spectrin beta, erythrocytic	The protein encoded by this locus functions in stability of erythrocyte membranes
TLR4	Toll like receptor 4	The protein encoded by this gene is a member of the Toll-like receptor (TLR) family which plays a fundamental role in pathogen recognition and activation of innate immunity. TLRs recognize pathogen-associated molecular patterns that are expressed on infectious agents, and mediate the production of cytokines necessary for the development of effective immunity

*Gene symbol, full gene name and its function are reported for the 10 genes listed in Table 3*.

To focus on SNVs with a potential effect of gene expression, the GTEx database was interrogated to filter variants which were eQTLs. Table 5 lists the 21 variants in five malaria genes and two variants in the ACE2 gene, that were finally selected: these are common variants, with an allele frequency in the studied population significantly correlated to COVID-19 cases (positively or negatively) and eQTLS in at least four tissues including lung.

**Table 5:** List of variants finally selected.

	**rs ID**	**Consequence**	**Gene symbol (input)**	**Spearman ρ**	**Spearman FDR**	**Captured alleles (Tagger)**	**eQTL genes associated**	**eQTLs slope lung**	**eQTL slope whole blood**
Positive correlation	rs72717040	Intronic	FCGR2A	0.48806	0.00431	rs17400517	FCGR2C, HSPA7	–	–
	rs17622656	Intronic	IRF1	0.51287	0.00208		SLC22A5	–	–
	rs8081547	Intronic	ADORA2B	0.55595	0.00049		CENPV	–	–
	rs7683365	Missense	GYPB	0.58510	0.00016	rs7662277, rs7666297, rs7666296, rs12499907, rs12499906, rs10002395, rs10025455, rs7661933	GYPE	–	–
Negative correlation	rs8176725	Intronic	ABO	−0.45895	0.00914		ABO	+	+
	rs6840234	Intronic	GYPB	−0.55690	0.00047	rs6857129, rs4835127, rs1473055, rs6537238, rs4835126, rs6816184	GYPE, FREM3	–	–
	rs4646120	Intronic	ACE2	−0.47397	0.00638		PIR	+	+
	rs1978124	Intronic	ACE2	−0.53219	0.00109		PIR	+	+

*The list of SNVs positively and negatively correlated with COVID-19 is reported in the upper and lower part of the table, respectively. These variants are strongly associated to COVID-19 (FDR < 0.01), are associated to eQTL in at least four tissues, including lung, and are driver-variants of other alleles. The first three columns represent the rsID, the consequence of the SNV on the gene, and the name of the malaria-associated gene where the variant is located. Spearman ρ and FDR are reported for each tag SNP. All the variants captured by the tag SNP are listed in “Captured alleles (Tagger)” column. The last three columns indicate the eQTL genes associated and the slope of the eQTL for lung and whole blood (+, positive; –, negative)*.

Excepted for the two variants in ACE2, all the other variants in the same gene were in LD and could be, therefore, considered together (Table 5). Only one missense variant was identified, named rs7683365 in the GYPB gene. All the others are intronic variants. The last three columns of Table 5 report the genes which are modulated by the variants and the effect on their expression in lung and whole blood. A concordance of the expression change between the two tissues has been noticed for all the variants except for rs8081547 in ADORA2B gene that increases the expression of the CENPV in lung but has an opposite effect in whole blood.

Among the variants whose frequency positively correlates with COVID-19 cases, we identified (i) two variants at FCGR2A gene which may potentially decrease the expression of FCGR2C and HSPA7, (ii) one variant in IRF1 gene which may upregulate SLC22A, (iii) a variant in ADORA2B which increases the mRNA expression of CENPV gene in lung, and (iv) 9 variants in GYPB (see below).

Variants with inverse correlation with COVID-19 cases include a variant in ABO which increases the expression of its transcript, and the two unrelated variants in ACE2 which may potentially increase the expression of PIR mRNA in both lung and blood.

Particular attention must be paid to the variants of GYPB. Actually, some of these variants positively correlate with COVID-19 cases and increase the expression of GYPE. Others that negatively correlate with COVID-19 determine a reduction in the transcripts of FREM and GYPE. This contradiction can be interpreted by assuming that variants positively correlated with the cases of disease induce an increase in protein expression, exerting their putative risk effect for COVID-19. Conversely, the negatively correlated, and therefore protective variants, exert their effect by decreasing FREM expression, rather than decreasing GYPE.

**Figure 2:**
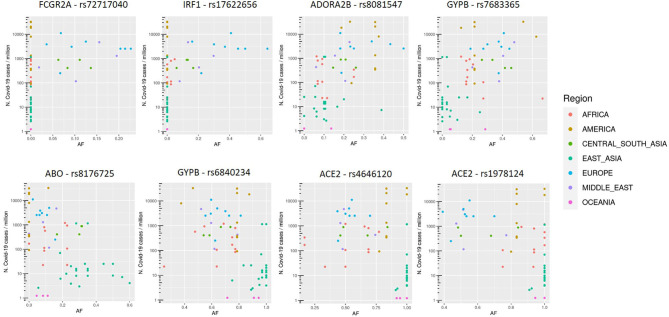
Allele frequency (AF) distribution in each population of selected variants, named with the rs number and gene symbol, in relation to the COVID-19 prevalence (n. COVID-19 cases/10^6^ inhabitants). Each dot is a population and colors are associated to specific geographic area, as reported in the legend.

SNVs in SPTB gene were not considered, since they resulted associated both positively and negatively with COVID-19 cases, all with eQTL with positive slopes. Variants thus selected are reported in Figure 2.

### Comparison With COVID-19 Cases From the Second Wave

The analysis of the correlation between Italian COVID-19 cases and deaths for malaria was also performed with the COVID-19 cases in the second wave (1st November 2020–15th February 2021), and we confirmed the correlation already observed (Spearman ρ = −0.53, *p*-value 1.2E-5). Similarly, the significant trend between allele frequencies and COVID-19 cases worldwide observed for the 8 SNVs in the first wave was corroborated by the data from the second wave (Spearman FDR ranges from 1.6E-04 to 3.3E-09).

## Discussion

In this study, we show that in the Italian population, the spread of SARS-CoV-2 in the first wave (until mid-July 2020) was more limited in the provinces where malaria was endemic at the beginning of the 20th century. Indeed, a highly significant inverse correlation emerged between COVID-19 cases and malaria mortality in 1910. A possible confounding factor could derive from the fact that the spread of SARS-CoV-2 is slightly greater in the northern Italian regions, while the malaria was endemic especially in the coasts of central and southern Italy. However, it is interesting to note that in some areas, such as in the provinces of Ferrara and Rovigo, located in the Po river delta and areas of malarial endemic, the spread of SARS-CoV-2 is much lower than in the neighboring provinces. These data have led to the hypothesis that some genetic variants, positively selected because of advantage against malaria, could also confer greater resistance to SARS-CoV-2 infection and thus explain the lower spread of the virus in the malarial areas.

A bioinformatic analysis of public worldwide WGS data led to the final identification of variants in 6 malaria related genes, whose frequency is significantly associated to the incidence of COVID-19 cases. Indeed, some of these genes have already been identified by case control studies as associated with COVID-19, while for the others a direct evidence for a role in SARS-CoV-2 infections or in the progression/severity of COVID-19 has never been reported in the literature.

We are aware that several factors could affect our observations. First, given the lack of genomic data from COVID-19 cases in malarial regions, we leveraged allele frequencies from healthy populations worldwide to highlight significant trends between MAF of variants in malarial genes and the prevalence of SARS-COV-2. In particular, variants from whole genome sequencing come from the Human Genome Diversity Project (HGDP) where, for each population, the approximate geographical place of origin was indicated by its latitude and longitude. Similarly, COVID-19 cases downloaded from the John Hopkins repository were annotated with latitude and longitude. We matched populations with similar coordinates in both datasets and, in cases where two populations were geographically very close, the COVID-19 or genetic data were aggregated in order to guarantee a geographical correspondence as reliable as possible. The final set used for downstream analysis consists of 46 populations for which both data were available. This approach does not take into account differences within countries (except for Italy and malaria) but we aimed at discovering global trends across populations worldwide. Although this is an indirect approach, several promising variants have emerged. Second, some of the subpopulations had a small sample size. Although they contribute to the estimate of the global genetic variability, the AF may not be representative of the population they belong to. The availability of multiple populations, and the use of a robust correlation, which is less sensitive to outliers, should mitigate this issue. Finally, in this study, we focused on the time frame corresponding to the first wave of pandemic (until mid-July 2020), however, the selected significant correlations were corroborated by the data from the second wave (Nov 2020-Feb 2021). Despite these issues, several variants passed all the filtering steps and were eventually selected.

In the following, we report on a possible link with SARS-CoV-2 infections, also in terms of molecular mechanisms and potential therapeutic options.

### ABO Blood Group

Several epidemiological analyses revealed that blood group O is associated with a lower risk of SARS-CoV infection (30). Using a cellular model of adhesion to investigate whether natural antibodies of the ABO system could block the S protein and ACE2 interaction, Guillon et al. have reported that the S protein/ACE2-dependent adhesion was specifically inhibited by either a monoclonal or human natural anti-A antibodies, indicating that these antibodies may block the interaction between the virus and its receptor, thereby providing protection. To fully appreciate the potential effect of the ABO polymorphism on the epidemiology of SARS-CoV, a mathematical model of the virus transmission dynamics that takes into account the protective effect of ABO natural antibodies was built. The model indicated that the ABO polymorphism could contribute to substantially reduce the virus transmission, affecting both the number of infected individuals and the kinetics of the epidemic (31).

These results gathered for SARS-CoV are consistent with recent observations with SARS-CoV-2. Li et al. have recently published “Association between ABO blood groups and risk of SARS-CoV-2 pneumonia” (32), an observation already reported by Zhao et al., and which have had a certain impact in the press (33). In both studies, the ABO blood groups distribution of patients with COVID-19 were compared to that of controls from the local populations. These studies show that blood group A is associated with an increased risk of infection, whereas group O is associated with a decreased risk. Recently, a genome-wide association study (GWAS) has confirmed a higher risk among persons with blood group A relative to those with other blood groups and a clear protective role for blood group O (34).

Histo-blood group ABO system transferase is an enzyme with glycosyltransferase activity, which is encoded by the ABO gene in humans. ABO determines the ABO blood group of an individual by modifying the oligosaccharides on cell surface glycoproteins. In our study, we observed that the intron variant rs8176725 of ABO determined an increase in histo-blood group ABO system transferase expression and correlated negatively with COVID-19 cases. This is consistent with the previous reported observations revealing that blood group O was associated with a low risk of SARS-CoV and SARS-CoV-2 infections and that ABO natural antibodies can block the interaction between S protein and ACE2.

Of interest, similar results were obtained for malaria infection. A meta-analysis study showed an increased odds of severe *P. Falciparum* infection among individuals with blood group A, B, AB, or non-O compared with blood group O. However, the difference in the level of *P. Falciparum* parasitaemia was not significant among individuals with blood group A or non-O compared with blood group O. Blood group A likely promotes increased severity of disease through mechanisms dependent on rosetting and cytoadhesion (35).

### Glycophorin B

Glycophorin A, B, and E (GYPA, GYPB, and GYPE) represent a small gene family localized on chromosome 4q28-q31 that encodes the major red cell membrane glycophorins, GPA and GPB, and GPE. Glycophorins play an important role in the invasion of red blood cells (RBCs) by malaria parasite. The glycophorin gene cluster has a complex pattern of gene conversion and structural variation, and selective pressure due to pathogens, including malaria, has contributed to induce diversity in this region (36). However, at present no data indicating a correlation between the GYPB rs6840234 variant and resistance to malaria are available. This intron-variant is characterized by a reduction in GYPE and FREM3 transcription. This finding is rather intriguing as the FREM3/GYPE is one of the most prominent examples of putative ancient balancing selection in a genome-wide analysis of haplotype sharing between humans and chimpanzees.

FREM3 (FRAS1-related extracellular matrix protein 3) is a gene located near GYPE and encodes an extracellular matrix protein that is required for maintaining the integrity of the skin epithelium and the differentiated state of renal epithelia. Although FREM3 apparently is not correlated with malaria and COVID-19, a recent study has demonstrated a link between a variant of FREM3 (rs186873296) and malaria (37). People carrying one copy of FREM3 rs186873296 allele have a reduced risk of severe malaria by about 40% (38). Moreover, the associations between sickle-cell trait and homozygosity for rs186873296 in FREM3 determines a phenotype with high hemoglobin concentration. The most likely reason for the high hemoglobin concentrations in cases with both these genotypes is that such children are protected from all forms of clinical malaria that result cumulatively in higher levels of anemia in children without these protective polymorphisms. Furthermore, rs186873296 FREM3 polymorphism correlates closely with the polymorphism that is specific to Dantu a hybrid gene comprising GYPA and GYPB, which encodes a blood antigen known as Dantu (Red blood cell tension protects against severe malaria in the Dantu blood group) (39). People with Dantu produce a hybrid molecule that consists of the extracellular domain of GYPB and the transmembrane plus intracellular domains of GYPA. The blood group variant Dantu provides 74% protection against all forms of severe malaria in homozygous individuals. This protection is due to a higher average tension in RBCs surface membrane which make them resistant to the parasite invasion.

If the role of GYPB as a protective factor against malarial infection, either directly or through FREM3, is sufficiently clarified, there are no data in the literature in relation to SARS-CoV-2 infection. GYPB rs6840234 decreases the expression of both glycophorin B and FREM3, and how this effect can protect against virus infection remains to be elucidated.

### Angiotensin Converting Enzyme 2

We observed two intron-variants of ACE2 gene that were negatively correlated with COVID-19 cases. These variants (rs4646120 and rs1978124) were able to increase the mRNA expression of PIR, a gene encoding the enzyme quercetin 2,3-dioxygenase, which is involved in the degradation of quercetin by a dioxygenase reaction. ACE2 and PIR are two genes located on X-chromosome and are co-regulated by “double elite” enhancers. The co-regulation of ACE2 and PIR represents an important step during SARS-CoV-2 infections. Actually, after interaction between SARS-CoV-2 and the ACE2 receptor, this complex is internalized with a marked ACE2 depletion in cell surface. ACE2 is an essential protein under feedback regulation to restore protein expression. Thus, ACE2 depletion is followed by an upregulation of ACE2 gene transcription. ACE2 gene lies in a co-regulated cluster with PIR and VEGFD, sharing “double-elite” enhancers, implying homeostatic host responses (40). ACE2 gene is sited on human X-chromosome between CLTRN and PIR genes, which is adjacent to VEGF-D. These genes share enhancers and other regulatory elements with ACE2. Thus, homeostatic responses attempting to restore expression of cell surface ACE2, during SARS-CoV-2 infections, can induce the upregulation of these genes. In particular, two enhancers of ACE2, GH0XJ015596 and GH0XJ015579, have double-elite interaction with the promoter of PIR and the promoter of VEGF-D (41). Thus, ACE2 upregulation is associated with increased expression of PIR and VEGF-D.

Quercetin 2,3-dioxygenase encoded by PIR converts the “antioxidant” quercetin to quinone, with a semiquinone radical intermediate, which reacts with oxygen to generate reactive oxygen species (ROS) superoxide O2·– and H2O2 (42). This causes a “ROS storm” which would account for a catastrophic deterioration in COVID-19 patients who are unable to suppress viral replication rapidly. Of interest, evidences indicate that quercetin is effective both for prophylaxis in high-risk populations and for the treatment of SARS-CoV-2 infections (43). The intron-variants rs4646120 and rs1978124 are characterized by high expression of PIR. Like to the ACE2 regulation during SARS-CoV-2 infection, overexpression of PIR may activate a negative feedback with reduction of the enhancers that co-regulate PIR and ACE2. The reduced expression of ACE2 would make people with these intronic variants less susceptible to SARS-CoV-2 infections.

### Interferon Regulatory Factor 1

We identified an intron variant in IRF1 gene (rs17622656) that positively correlated with COVID-19. Interferon regulatory factor 1 (IRF1) was the first member of the interferon regulatory transcription factor family identified. IRF1 regulates expression of target genes by binding to an interferon stimulated response element (ISRE) in their promoters. Several reports suggest that IFN-α and IFN-γ can co-operatively inhibit some virus replication including SARS-CoV, and IRF-1 plays a key role in this process (44). Conversely, the intron variant IRF-1 rs17622656 positively correlated with COVID-19 cases. This variant is characterized by a reduced expression of SLC22A5 (OCTN2) gene. This gene encodes a protein involved in the active cellular uptake of carnitine.

The observation that leukocytes, including monocytes and lymphocytes, are enriched in carnitines first suggested that carnitine may regulate the immune response (45). A reduced pool of carnitines has been demonstrated in either serum or tissues, or both, from patients with unregulated and/or impaired immune responses. Carnitine deficiency in peripheral blood lymphocytes and monocytes is common among HIV patients, and this is linked to a wide spectrum of mechanisms ranging from malabsorption to defective synthesis (46). Clinical studies have demonstrated that in HIV patients carnitine therapy in combination with standard antiretroviral treatment could have a greater improvement in CD4 counts (47). Therefore, the positive correlation between COVID-19 cases and the intron variant IRF1 rs17622656 could lead to a reduced uptake of carnitine and to an impaired immune response against SARS-CoV-2.

Recently, particular attention has been focused on IFN genes and those that regulate them. It is unknown whether rs17622656 negatively regulates IRF1, which in turn would act downstream on the IRF genes. If this effect was demonstrated, the positive correlation with COVID-19 cases would be explainable. In line with this hypothesis, there are findings demonstrating the presence of mutations in type I IFN-related genes in patients with life-threatening COVID-19 pneumonia (48).

### Adenosine A2b Receptor

ADORA2B encodes for an adenosine receptor. We found 2 introns variants (rs4646120 and rs1978124) of ADORA2B which correlated with COVID-19. These variants decrease protein expression in the lung. The activity of this adenosine receptor is mediated by G proteins which activate adenylyl cyclase. Adenosine is an endogenous ligand for four different adenosine receptor subtypes (AdoRA1, AdoRA2A, AdoRA2B, and AdoRA3). Increased concentrations of adenosine were found in ascites of MethA sarcoma or in culture medium of 3LL Lewis lung carcinoma growing under hypoxic conditions. Adenosine and its analogs efficiently inhibited the cytotoxic activity of LAK cells. In fact, intratumor adenosine impairs the ability of lymphokine-activated killer (LAK) cells to kill tumor cells. It is possible to hypothesize that a decrease in ADOR2B may decrease the NK response toward cells infected with SARS-CoV-2, although there are no experimental studies that can confirm this hypothesis (49).

Alternatively, ADORA2B could directly affect lung function. In fact, there are many instances where acute lung injury (ALI) resolves spontaneously through endogenous pathways that help to control excessive lung inflammation. Previous studies have implicated the extracellular signaling molecule adenosine and signaling events through the A2B adenosine receptor in lung protection. In this context, alveolar epithelial A2B adenosine receptor signaling contributes to lung protection. Thus, variants that induce a decrease in ADORA2B in the lung may be risk factors for the evolution of SARS-CoV-2 infection (50).

### Fc Fragment of IgG Receptor IIa

We found that the frequency of intron variants rs72717040 and rs17400517 of FCGR2A correlated with the frequency of COVID-19. This gene encodes one member of a family of immunoglobulin Fc receptor genes found on the surface of many immune response cells. The protein encoded by this gene is a cell surface receptor found on phagocytic cells such as macrophages and neutrophils and is involved in the process of phagocytosis and clearing of immune complexes. It has also been associated with severe malaria in Gambia and in Kenia (51, 52).

The polymorphisms of FCGR2A exert a downregulation effect for both the FCGR2C and HSPA7 genes. The protein encoded by the first gene is again a receptor for the Fc region of complexed immunoglobulins gamma and is involved in a variety of effector and regulatory functions such as phagocytosis of immune complexes and modulation of antibody production by B-cells. HSPA7 is a pseudogene, member of Heat Shock Protein Family A (Hsp70). It is unclear how a decrease in FCGR2C can affect SARS-CoV-2 infection, and there is no association data between this gene and COVID-19. It is possible to hypothesize that its decrease in the lung and in phagocytic cells could reduce the effectiveness of the immune response to the virus.

It is also interesting to note that other polymorphisms in FCGR2A have been associated with Kawasaki disease (53, 54). This disease has been addressed in more than 200 studies published in the last 6 months: an unusually high incidence of Kawasaki disease in children was reported and in many of them IgG antibodies for SARS-CoV-2 were detected, suggesting an association between the SARS-CoV-2 and this syndrome in children. Although Kawasaki disease-like syndromes have previously been linked to other viral infections, these patients showed higher levels of pro-inflammatory markers than other cohorts, which may reflect a particularly strong immunological reaction to SARS-CoV-2 (55, 56). In brief, notwithstanding a direct link between FCGR2A and COVID-19 has not been identified yes, it appears quite intriguing that this gene has previously been associated to Kawasaki disease, which in turn has been associated to COVID-19.

## Conclusions

The present study demonstrates that some genetic variants selected to be protective against malaria may also play a role in the severity of SARS-CoV-2 infections. After identifying the genes known to be associated with malaria, we focused on those variants related to the frequency of COVID-19, both directly and inversely. After further skimming, we pinpointed six genes, representing a possible link between malaria and COVID-19. Indeed, for some of these, there is already direct evidence of implication with SARS-CoV-2 infection or with the evolution of COVID-19, such as ABO (blood groups) and ACE2 (a key human receptor for SARS-CoV-2). For other genes, there are interesting hypotheses for their role on COVID-19, as in IRF1 and ADORA2B. Finally, for the genes GYPB and FCGR2A, further investigations are needed to confirm an association with COVID-19 along with molecular studies to shed light on the mechanisms responsible for higher/lower incidence of SARS-CoV-2 infections.

Among the genetic outcomes, the one related to the uptake of carnitine particularly attracted our attention. Carnitine is actually responsible for maintaining a high level of the immune response, and a lower level of this amino acid can negatively impact lymphocytes and monocytes. Therefore, we point to L-carnitine (or its prodrug acetyl-L-carnitine) as a possible dietary supplement to improve the immune response and combat/prevent COVID-19. A clinical trial to study the role of carnitine in addition to the standard of care has recently been launched.

We want to conclude that the COVID-19 pandemic was rather brutal, particularly in the initial steps, forcing the health systems to concentrate all energy on the hospitals' emergency. This hampered the possibility to plan the collection of biological samples from COVID-19 patients for subsequent genomics and/or other analyses. Furthermore, to carry out a malaria-COVID-19 correlation study, one would have needed to find biological samples from malarial areas, along with clinical information on the malarial patient's history. All these steps are currently planned and will represent the subject matter of subsequent studies in the field (which are currently ongoing), where the present genetic variants will be confirmed. New ones will likely be disclosed to shed further light on the biological and genetic mechanisms of this complex infection, responsible for the current worldwide pandemic. Finally, possible links may emerge among variants in the human and viral genome, as intensive programs of high throughput sequencing are currently ongoing worldwide.

## Summary

Despite being rather different, malaria and COVID-19 may show some commonalities, mainly related to genetic variants present in populations living in malaria-endemic regions. In particular, starting from the evidence that in Italy, the areas with the lowest number of COVID-19 cases were those with the highest incidence of malaria, we here analyze genomic data from a May 2020 database. We filter the database for ACE2 and variants that are connected to malaria, pointing eventually to 47 genes. Further focusing only on those variants statistically relevant, we ultimately identify 6 genes that could be responsible for major susceptibility (or major resistance) to malaria. Surprisingly enough, these variants are also statistically related to higher/lower incidence/severity of SARS-CoV-2 infections. Among others, we identified genetic variants connected to blood group O, to a reduced expression of the ACE2 receptor (the entry point of SARS-CoV-2 into the human body), and to a decreased uptake of carnitine, an amino acid that plays a key role in the immune system response to infections. In conclusion, this study sheds further light on the genetic and molecular mechanisms responsible for the high/low impact of COVID-19. It points to a possible dietary supplement (L-carnitine) as a therapeutic adjuvant in treating SARS-CoV-2 infections.

## Data Availability Statement

Publicly available datasets were analyzed in this study. These data can be found at: ftp://ngs.sanger.ac.uk/production/hgdp (WGS data) and https://github.com/CSSEGISandData/COVID-19/tree/master/csse_covid_19_data/csse_covid_19_daily_reports (COVID-19 data).

## Author Contributions

MR drafted the manuscript, performed the analysis, and analyzed the results. PU designed the pipeline, performed the bioinformatic analysis, and analyzed the results. AA and MT analyzed the results. AC conceived the study, analyzed the results, and wrote the manuscript. All authors contributed to manuscript revision, read, and approved the submitted version.

## Conflict of Interest

The authors declare that the research was conducted in the absence of any commercial or financial relationships that could be construed as a potential conflict of interest.
